# Supplementary material for: Social Communication in Mice – Are There Optimal Cage Conditions?
Source: PLoS One. 2015 Mar 25;10(3):e0121802. doi: 10.1371/journal.pone.0121802 (PMC4373896; doi:10.1371/journal.pone.0121802)
Supplement: S1 Table — Information about the habituation time, the cage shape, the duration of the test, the background of the wild-type strain studied is compiled. Selected articles: [13,16–19,22,30–49]. (PDF) [file pone.0121802.s001.pdf]

| model | publication | test | habituation | cage size | cage area | observation time | time of interaction | time of interaction (%) | background & sex |
|---|---|---|---|---|---|---|---|---|---|
| **Adult same-sex social interactions** | | | | | | | | | |
| *Mecp2* 308/Y (truncat | Pearson et al. (2012) | free social interactions with 2 adults (interactor: C57BL/6J) | no | 7 x 14 x 30 cm | 98 $cm^2$ | NA | per cct types | per cct types | C57BL/6J, males |
| *Sept5*-KO | Harper et al. (2012) | free social interactions with 2 adults (pairs of 1 mutant and 1 C57BL/6 mouse) | both 30 min in a fresh home cage (different from test cage) | NA (27 x 16 x 12.5 cm?) | 432 $cm^2$? | 5 min + 5 min separated by 30 min | 30 s (trial 1) | 10% (trial 1) | C57BL/6J, males |
| BTBR T+tf/J | Scattoni et al. (2010) | free social interactions with 2 adults | individual housing for 5 days | 36.9 x 15.6 x 13.2 cm | 575.64 $cm^2$ | 3 min | 67 s (m), 25 s (f) | 37.2% (m), 13.9% (f) | C57BL/6J, males & females |
| C57BL/6N | Hammerschmidt et al. (2012) | free social interactions with 2 adults | 60 s | 36 x 20 cm? | 720 $cm^2$? | 3 min | NA | NA | C57BL/6N, males & females |
| *Tsc1*-HZ | Goorden et al. (2007) | free social interactions with 2 adults (2 interactors: 129P2-C57BL/6) | 15 min | 45 x 20 cm | 900 $cm^2$ | 2 min + 2 min + 2 min | 58% (trial 1) | 58% (trial 1) | C57BL/6J*C57BL/6N, females |
| BTBR T+tf/J | Silverman et al. (2012) | free social interactions with 2 adults (interactor: 129/SvImJ) | no hab tot the test cage (1h individual housing before the test) | Noldus PhenoTyper 3000 arena (30 x 30 x 30 cm) | 900 $cm^2$ | 10 min | 150 s | 25% | C57BL/6J, males? |
| *Nlgn3*-KO | Radyushkin et al. (2009) | free social interactions with 2 adults (same genotype) | hab to the test cage 10-min for 2 days before the test | neutral cage (30 x 30 x 30 cm ) | 900 $cm^2$ | 10 min | 75 s | 12.5% | C57BL/6N, males |
| *Nlgn4*-KO | Jamain et al. (2008) | free social interactions with 2 adults (same genotype) | hab to the test cage 10-min for 2 days before the test | neutral cage (gray Plexiglas box, 30 x 30 x 30 cm) | 900 $cm^2$ | 10 min | 115 s | 19.2% | C57BL/6J, males |
| *Nlgn4*-KO | El-Kordi et al. (2013) | free social interactions with 2 adults (same genotype) | 10 min on two consecutive days before testing | 30 x 30 x 30 cm | 900 $cm^2$ | 10 min | 87 s (m), 66 s (f) | 14.5% (m), 11% s (f) | C57BL/6J, males & females |
| *Shank3*-KO | Wang et al. (2011) | free social interactions with 2 adults (interactor: C3H) | 5 min (in a partitionned cage, after 14 days of individual housing) | 48 x 26 x 20 cm | 1248 $cm^2$ | NA | 21 s (m), 29 s (f) | NA | C57BL/6J, males & females |
| *Nlgn4*-KO | Ey et al. (2012) | free social interactions with 2 adults (interactor: WT) | 30 min | 50 x 25 x 30 cm | 1250 $cm^2$ | 4 min | NA | NA | C57BL/6J, males & females |
| *Shank2*-KO | Schmeisser et al. (2012) | free social interactions with 2 adults (interactor: WT) | 30 min | 50 x 25 x 30 cm | 1250 $cm^2$ | 4 min | 85 s (m), 135 s (f) | 35.4% (m), 56.3% (f) | C57BL/6, males & females |
| *Shank3*-KO | Peça et al. (2011) | free social interactions with 2 adults (interactor: WT) | 1h in the room | 40 x 40 x 30 cm | 1600 $cm^2$ | 10 min | 86 s | 14.3% | hybrid background, males |
| *Fmr1-KO & TgN1F* | Dahlhaus & El-Husseini (2009) | free social interactions with 2 adults (interactor: WT) | 10 min (in a cage separated in 2) | 40 x 60 cm | 2400 $cm^2$ | 10 min | relative changes | relative changes | C57BL/6, males |
| C57BL/6J | Chabout et al. (2012) | free social interactions with 2 adults (same genotype) | 30 min | 50 x 30 x 30 cm | 1500 $cm^2$ | 4 min | NA | NA | C57BL/6J, males |
| Dupl 15q11-13 (ch 7 in mice) | Nakatani et al. (2009) | free social interactions with 2 adults (same genotype) | no hab in the test cage | 40 x 40 x 30 cm | 1600 $cm^2$ | 10 min | 133 s | 22.2% | 129SvJ x C57BL/6, males |
| | | | | | | | | | |
| *Mecp2* 308/Y (truncation) | Moretti et al. (2004) | resident-intruder test (intruder=129S6/SvEv) | resident in its home cage | 28.5 x 17.5 x 12 cm | 498.75 $cm^2$ | 10 min (3 consecutive days) | 48% of time | 48% | 129SvEv, males |
| *Mecp2* conditional KO (in hypothalamus) | Fyffe et al. (2008) | resident-intruder test (intruder=C57BL/6) | resident in its home cage for 2 weeks | NA (45 x 24 x 17 cm?) | 1080 $cm^2$? | 10 min | per cct types | per cct types | 129SvEv x FVB, males |
| | | | | | | | | | |
| **Juvenile same-sex social interactions** | | | | | | | | | |
| *Nrxn1α*-KO | Etherton et al. (2009) | free social interactions with a juvenile | no hab in the test cage (only to the room) | same size as home cage (27 cm x 16 cm x 12 cm?; from Kogan et al. 2000) | 432 $cm^2$? | 2 min | 38 s | 31.6% | NA, males & females |
| *Mecp2* 308/Y (truncation) | Moretti et al. (2004) | free social interactions with a juvenile (129S6/SvEv) | 15 min | 28.5 x 17.5 x 12 cm | 498.75 $cm^2$ | 2 min | 70 s | 58.3% | 129SvEv, males |
| *Mecp2* 308/Y (truncation) | Moretti et al. (2006) | free social interactions with a juvenile (C57BL/6J) | 15 min | 28.5 x 17.5 x 12 cm | 498.75 $cm^2$ | 2 min | 78 s | 65% | 129SvEv, males |
| *Nlgn4*-KO | Ey et al. (2012) | free social interactions with 2 juveniles (interactor: C57BL/6J) | 1h in isolation before testing (in a different cage) | Noldus PhenoTyper arena (25 x 25 x 35 cm) | 625 $cm^2$ | 10 min | per cct types | per cct types | C57BL/6J, males & females |
| *Nlgn3*-KI (R451C) | Chadman et al. (2008) | juvenile social play | 10 min of habituation one day before testing | Noldus PhenoTyper arena (30 x 30 x 30 cm) | 900 $cm^2$ | 30 min observation | per cct types | per cct types | C57BL/6J, males & females |
| BTBR T+tf/J | MacFarlane et al. (2008) | juvenile social play | 10 min | Noldus PhenoTyper arena (30 x 30 x 30 cm) | 900 $cm^2$ | 30 min | per cct types | per cct types | C57BL/6J, males & females |
| *Shank1*-KO | Silverman et al. (2011) | juvenile social play | no (individual housing for 1h) | Noldus PhenoTyper 3000 arena (30 x 30 x 30 cm) | 900 $cm^2$ | 10 min | per cct types | per cct types | C57BL/6J*129SvJae, males & females |
| *engrailed-2*-KO | Brielmaier et al. (2012) | free social interactions with 2 juveniles (interactor: C57BL/6J) | housing in individual standard mouse cages 1h before testing | Noldus PhenoTyper arena (30 x 30 x 30 cm) | 900 $cm^2$ | 10 min | per cct types | per cct types | 129S2/SvPas |
| *Shank3*-KO | Yang et al. (2012) | free social interactions with 2 juveniles (interactor: C57BL/6J) | no (individual housing for 1h) | Noldus PhenoTyper 3000 arena (25 x 25 x 35 cm) | 900 $cm^2$ | 10 min | per cct types | per cct types | C57BL/6, males & females |
| | | | | | | | | | |
| **Male-female interactions** | | | | | | | | | |
| C57BL/6J | Yang et al. (2013) | male-female interactions | no | 35 x 14 x 36 cm | 490 $cm^2$ | 5 min + 3 min separated by 3 min | 18 s / min | 30% | C57BL/6J, males |
| BTBR T+tf/J | Scattoni et al. (2010) | male-female interactions | individual housing for 5 days | 36.9 x 15.6 x 13.2 cm | 575.64 $cm^2$ | 5 min | 45 s | 15% | C57BL/6J, males & females |
| *Fmr1*-KO | Rotschafer et al. (2012) | male-female interactions (same genotype) | no hab in the test cage | 28.8 x 21.6 x 28.8 cm | 622.1 $cm^2$ | until mating occurred (or max of 20 min) | NA | NA | FVB/129P2, males |
| C57BL/6N | Hammerschmidt et al. (2012) | male-female interactions | 60 s | 36 x 20 cm? | 720 $cm^2$? | 3 min | NA | NA | C57BL/6N, males |
| *Nlgn4*-KO | Ey et al. (2012) | male-female interactions (B6 female) | 10 min | 50 x 25 x 30 cm | 1250 $cm^2$ | 3 min | 130 s | 72.2% | C57BL/6J, males |
| *Shank2*-KO | Schmeisser et al. (2012) | male-female interactions (B6 female) | 10 min | 50 x 25 x 30 cm | 1250 $cm^2$ | 3 min | 120 s | 66.7% | C57BL/6, males |
