# Supplementary material for: Social Communication in Mice – Are There Optimal Cage Conditions?
Source: PLoS One. 2015 Mar 25;10(3):e0121802. doi: 10.1371/journal.pone.0121802 (PMC4373896; doi:10.1371/journal.pone.0121802)
Supplement: S2 Table — Comparisons were made to identify the effect of habituation time and cage shape/size. (PDF) [file pone.0121802.s002.pdf]

	4-min				8-min			
**time in contact**								
Habituation time			Mann-Whitney U-test				Mann-Whitney U-test	
			20-min vs 30-min				*20-min vs 30-min*	
rectangle			W = 33, p = 0.959				W = 31, p = 0.959	
round			W = 10, p = 0.021				W = 3, p = 0.001	
square			W = 28, p = 0.721				W = 30, p = 0.879	
Cage shape	Kruskal-Wallis rank sum test		Mann-Whitney U-test		Kruskal-Wallis rank sum test		Mann-Whitney U-test	
		rectangle vs round	*round vs square*	*rectangle vs square*		*rectangle vs round*	*round vs square*	*rectangle vs square*
20-min habituation	X = 5.055, df = 2, p = 0.080	NA	NA	NA	X = 4.085, df = 2, p = 0.130	NA	NA	NA
30-min habituation	X = 0.455, df = 2, p = 0.797	NA	NA	NA	X = 1.095, df = 2, p = 0.578	NA	NA	NA
**nose-to-nose contact (duration)**								
Habituation time			Mann-Whitney U-test				Mann-Whitney U-test	
			20-min vs 30-min				*20-min vs 30-min*	
rectangle			W = 29, p = 0.798				W = 33.5, p = 0.916	
round			W = 6, p = 0.005				W = 9, p = 0.015	
square			W = 32, p = 1				W = 29, p = 0.798	
Cage shape	Kruskal-Wallis rank sum test		Mann-Whitney U-test		Kruskal-Wallis rank sum test		Mann-Whitney U-test	
		rectangle vs round	*round vs square*	*rectangle vs square*		*rectangle vs round*	*round vs square*	*rectangle vs square*
20-min habituation	X = 1.085, df = 2, p = 0.581	NA	NA	NA	X = 0.992, df = 2, p = 0.609	NA	NA	NA
30-min habituation	X = 3.505, df = 2, p = 0.173	NA	NA	NA	X = 2.885, df = 2, p = 0.236	NA	NA	NA
**oro-genital contact (duration) from the occupant**								
Habituation time			Mann-Whitney U-test				Mann-Whitney U-test	
			20-min vs 30-min				*20-min vs 30-min*	
rectangle			W = 37, p = 0.645				W = 31.5, p = 1	
round			W = 15, p = 0.083				W = 6, p = 0.005	
square			W = 28, p = 0.721				W = 30, p = 0.879	
Cage shape	Kruskal-Wallis rank sum test		Mann-Whitney U-test		Kruskal-Wallis rank sum test		Mann-Whitney U-test	
		rectangle vs round	*round vs square*	*rectangle vs square*		*rectangle vs round*	*round vs square*	*rectangle vs square*
20-min habituation	X = 5.46, df = 2, p = 0.065	NA	NA	NA	X = 4.781, df = 2, p = 0.092	NA	NA	NA
30-min habituation	X = 0.945, df = 2, p = 0.623	NA	NA	NA	X = 1.755, df = 2, p = 0.416	NA	NA	NA
**oro-genital contact (duration) from the new-comer**								
Habituation time			Mann-Whitney U-test				Mann-Whitney U-test	
			20-min vs 30-min				*20-min vs 30-min*	
rectangle			W = 24, p = 0.442				W = 21, p = 0.279	
round			W = 30, p = 0.879				W = 34, p = 0.875	
square			W = 25, p = 0.505				W = 29, p = 0.798	
Cage shape	Kruskal-Wallis rank sum test		Mann-Whitney U-test		Kruskal-Wallis rank sum test		Mann-Whitney U-test	
		rectangle vs round	*round vs square*	*rectangle vs square*		*rectangle vs round*	*round vs square*	*rectangle vs square*
20-min habituation	X = 1.685, df = 2, p = 0.431	NA	NA	NA	X = 5.497, df = 2, p = 0.064	NA	NA	NA
30-min habituation	X = 2.78, df = 2, p = 0.249	NA	NA	NA	X = 1.900, df = 2, p = 0.387	NA	NA	NA
**follow behaviour (duration)**								
Habituation time			Mann-Whitney U-test				Mann-Whitney U-test	
			20-min vs 30-min				*20-min vs 30-min*	
rectangle			W = 30, p = 0.879				W = 31, p = 0.959	
round			W = 39, p = 0.505				W = 38, p = 0.574	
square			W = 18, p = 0.161				W = 18, p = 0.161	
Cage shape	Kruskal-Wallis rank sum test		Mann-Whitney U-test		Kruskal-Wallis rank sum test		Mann-Whitney U-test	
		rectangle vs round	*round vs square*	*rectangle vs square*		*rectangle vs round*	*round vs square*	*rectangle vs square*
20-min habituation	X =2.547, df = 2, p = 0.280	NA	NA	NA	X = 0.965, df = 2, p = 0.617	NA	NA	NA
30-min habituation	X = 5.415, df = 2, p = 0.067	NA	NA	NA	X = 5.345, df = 2, p = 0.069	NA	NA	NA
**occupant approaches new-comer (occurrences)**								
Habituation time			Mann-Whitney U-test				Mann-Whitney U-test	
			20-min vs 30-min				*20-min vs 30-min*	
rectangle			W = 21.5, p = 0.292				W = 24.5, p = 0.462	
round			W = 17.5, p = 0.141				W = 21, p = 0.279	
square			W = 35, p = 0.793				W = 30.5, p = 0.916	
Cage shape	Kruskal-Wallis rank sum test		Mann-Whitney U-test		Kruskal-Wallis rank sum test		Mann-Whitney U-test	
		rectangle vs round	*round vs square*	*rectangle vs square*		*rectangle vs round*	*round vs square*	*rectangle vs square*
20-min habituation	X = 0.452, df = 2, p = 0.798	NA	NA	NA	X = 1.905, df = 2, p = 0.386	NA	NA	NA
30-min habituation	X = 2.324, df = 2, p = 0.313	NA	NA	NA	X = 2.143, df = 2, p = 0.343	NA	NA	NA
**new-comer approaches occupant (occurrences)**								
Habituation time			Mann-Whitney U-test				Mann-Whitney U-test	
			20-min vs 30-min				*20-min vs 30-min*	
rectangle			W = 34.5, p = 0.834				W = 35.5, p = 0.752	
round			W = 30.5, p = 0.916				W = 30.5, p = 0.916	
square			W = 41, p = 0.370				W = 45.5, p = 0.171	
Cage shape	Kruskal-Wallis rank sum test		Mann-Whitney U-test		Kruskal-Wallis rank sum test		Mann-Whitney U-test	
		rectangle vs round	*round vs square*	*rectangle vs square*		*rectangle vs round*	*round vs square*	*rectangle vs square*
20-min habituation	X = 0.117, df = 2, p = 0.943	NA	NA	NA	X = 0.905, df = 2, p = 0.636	NA	NA	NA
30-min habituation	X = 1.043, df = 2, p = 0.594	NA	NA	NA	X = 3.723, df = 2, p = 0.156	NA	NA	NA
**occupant escapes from new-comer (occurrences)**								
Habituation time			Mann-Whitney U-test				Mann-Whitney U-test	
			20-min vs 30-min				*20-min vs 30-min*	
rectangle			W = 42, p = 0.318				W = 30, p = 0.875	
round			W = 8, p = 0.013				W = 12, p = 0.040	
square			W = 55.5, p = 0.016				W = 52, p = 0.040	
Cage shape	Kruskal-Wallis rank sum test		Mann-Whitney U-test		Kruskal-Wallis rank sum test		Mann-Whitney U-test	
		rectangle vs round	*round vs square*	*rectangle vs square*		*rectangle vs round*	*round vs square*	*rectangle vs square*
20-min habituation	X = 2.312, df = 2, p = 0.315	NA	NA	NA	X = 5.462, df = 2, p = 0.065	NA	NA	NA
30-min habituation	X = 11.667, df = 2, p = 0.003	W = 12, p = 0.040	W = 60.5, p = 0.003	W = 51.5, p = 0.046	X = 8.562, df = 2, p = 0.014	W = 34, p = 0.875	W = 53.5, p = 0.027	W = 58, p = 0.007
**new-comer escapes from occupant (occurrences)**								
Habituation time			Mann-Whitney U-test				Mann-Whitney U-test	
			20-min vs 30-min				*20-min vs 30-min*	
rectangle			W = 15.5, p = 0.092				W = 21.5, p = 0.293	
round			W = 35.5, p = 0.753				W = 28, p = 0.713	
square			W = 19.5, p = 0.207				W = 17.5, p = 0.140	
Cage shape	Kruskal-Wallis rank sum test		Mann-Whitney U-test		Kruskal-Wallis rank sum test		Mann-Whitney U-test	
		rectangle vs round	*round vs square*	*rectangle vs square*		*rectangle vs round*	*round vs square*	*rectangle vs square*
20-min habituation	X = 0.499, df = 2, p = 0.779	NA	NA	NA	X = 0.857, df = 2, p = 0.652	NA	NA	NA
30-min habituation	X = 1.100, df = 2, p = 0.577	NA	NA	NA	X = 0.420, df = 2, p = 0.810	NA	NA	NA
**occupant approaches new-comer & new-comer escapes (occurrences)**								
Habituation time			Mann-Whitney U-test				Mann-Whitney U-test	
			20-min vs 30-min				*20-min vs 30-min*	
rectangle			W = 16.5, p = 0.115				W = 22, p = 0.315	
round			W = 33.5, p = 0.916				W = 32, p = 1	
square			W = 16, p = 0.1				W = 14.5, p = 0.073	
Cage shape	Kruskal-Wallis rank sum test		Mann-Whitney U-test		Kruskal-Wallis rank sum test		Mann-Whitney U-test	
		rectangle vs round	*round vs square*	*rectangle vs square*		*rectangle vs round*	*round vs square*	*rectangle vs square*
20-min habituation	X = 0.704, df = 2, p = 0.703	NA	NA	NA	X = 0.969, df = 2, p = 0.616	NA	NA	NA
30-min habituation	X =1.421, df = 2, p = 0.492	NA	NA	NA	X = 1.568, df = 2, p = 0.457	NA	NA	NA

new-comer approaches occupant & occupant escapes (occurrences)								
Habituation time			Mann-Whitney U-test				Mann-Whitney U-test	
			20-min vs 30-min				*20-min vs 30-min*	
rectangle			W = 32.5, p = 1				W = 39.5, p = 0.460	
round			W = 33, p = 0.958				W = 28.5, p = 0.751	
square			W = 47, p = 0.124				W = 49.5, p = 0.073	
Cage shape	Kruskal-Wallis rank sum test		Mann-Whitney U-test		Kruskal-Wallis rank sum test		Mann-Whitney U-test	
		rectangle vs round	*round vs square*	*rectangle vs square*		*rectangle vs round*	*round vs square*	*rectangle vs square*
20-min habituation	X = 1.232, df = 2, p = 0.540	NA	NA	NA	X = 1.554, df = 2, p = 0.460	NA	NA	NA
30-min habituation	X = 1.317, df = 2, p = 0.518	NA	NA	NA	X = 2.287, df = 2, p = 0.319	NA	NA	NA

occupant approaches new-comer & occupant escapes (occurrences)								
Habituation time			Mann-Whitney U-test				Mann-Whitney U-test	
			20-min vs 30-min				*20-min vs 30-min*	
rectangle			W = 35, p = 0.792				W = 30.5, p = 0.916	
round			W = 15, p = 0.081				W = 12.5, p = 0.046	
square			W = 59.5, p = 0.004				W = 53.5, p = 0.027	
Cage shape	Kruskal-Wallis rank sum test		Mann-Whitney U-test		Kruskal-Wallis rank sum test		Mann-Whitney U-test	
		rectangle vs round	*round vs square*	*rectangle vs square*		*rectangle vs round*	*round vs square*	*rectangle vs square*
20-min habituation	X = 3.478, df = 2, p = 0.176	NA	NA	NA	X = 4.805, df = 2, p = 0.091	NA	NA	NA
30-min habituation	X = 6.329, df = 2, p = 0.042	W = 23, p = 0.370	W = 54, p = 0.023	W = 48.5, p = 0.090	X = 5.857, df = 2, p = 0.053	NA	NA	NA

new-comer approaches occupant & new-comer escapes (occurrences)								
Habituation time			Mann-Whitney U-test				Mann-Whitney U-test	
			20-min vs 30-min				*20-min vs 30-min*	
rectangle			W = 20.5, p = 0.241				W = 28, p = 0.712	
round			W = 31, p = 0.957				W = 27, p = 0.634	
square			W = 32, p = 1				W = 32.5, p = 1	
Cage shape	Kruskal-Wallis rank sum test		Mann-Whitney U-test		Kruskal-Wallis rank sum test		Mann-Whitney U-test	
		rectangle vs round	*round vs square*	*rectangle vs square*		*rectangle vs round*	*round vs square*	*rectangle vs square*
20-min habituation	X = 0.099, df = 2, p = 0.952	NA	NA	NA	X = 1.172, df = 2, p = 0.557	NA	NA	NA
30-min habituation	X = 2.714, df = 2, p = 0.257	NA	NA	NA	X = 2.030, df = 2, p = 0.363	NA	NA	NA

new-comer in vision field of occupant (duration)								
Habituation time			Mann-Whitney U-test				Mann-Whitney U-test	
			20-min vs 30-min				*20-min vs 30-min*	
rectangle			W = 28, p = 0.721				W = 29, p = 0.798	
round			W = 51, p = 0.049				W = 64, p < 0.001	
square			W = 27, p = 0.645				W = 35, p = 0.798	
Cage shape	Kruskal-Wallis rank sum test		Mann-Whitney U-test		Kruskal-Wallis rank sum test		Mann-Whitney U-test	
		rectangle vs round	*round vs square*	*rectangle vs square*		*rectangle vs round*	*round vs square*	*rectangle vs square*
20-min habituation	X = 4.995, df = 2, p = 0.082	NA	NA	NA	X = 8.135, df = 2, p = 0.017	W = 5, p = 0.003	W = 48, p = 0.105	W = 22, p = 0.328
30-min habituation	X = 1.815, df = 2, p = 0.404	NA	NA	NA	X = 2.205, df = 2, p = 0.332	NA	NA	NA

occupant in vision-field of new-comer (duration)								
Habituation time			Mann-Whitney U-test				Mann-Whitney U-test	
			20-min vs 30-min				*20-min vs 30-min*	
rectangle			W = 34, p = 0.879				W = 29, p = 0.798	
round			W = 49, p = 0.083				W = 60, p = 0.002	
square			W = 35, p = 0.798				W = 36, p = 0.721	
Cage shape	Kruskal-Wallis rank sum test		Mann-Whitney U-test		Kruskal-Wallis rank sum test		Mann-Whitney U-test	
		rectangle vs round	*round vs square*	*rectangle vs square*		*rectangle vs round*	*round vs square*	*rectangle vs square*
20-min habituation	X = 4.145, df = 2, p = 0.126	NA	NA	NA	X = 7.265, df = 2, p = 0.026	W = 11, p = 0.028	W = 55, p = 0.015	W = 32, p = 1
30-min habituation	X = 0.455, df = 2, p = 0.797	NA	NA	NA	X = 0.665, df = 2, p = 0.717	NA	NA	NA

occupant stops (duration)								
Habituation time			Mann-Whitney U-test				Mann-Whitney U-test	
			20-min vs 30-min				*20-min vs 30-min*	
rectangle			W = 33, p = 0.959				W = 30, p = 0.879	
round			W = 45, p = 0.195				W = 43, p = 0.279	
square			W = 38.5, p = 0.528				W = 34, p = 0.879	
Cage shape	Kruskal-Wallis rank sum test		Mann-Whitney U-test		Kruskal-Wallis rank sum test		Mann-Whitney U-test	
		rectangle vs round	*round vs square*	*rectangle vs square*		*rectangle vs round*	*round vs square*	*rectangle vs square*
20-min habituation	X = 0.555, df = 2, p = 0.758	NA	NA	NA	X = 3.02, df = 2, p = 0.221	NA	NA	NA
30-min habituation	X = 3.515, df = 2, p = 0.173	NA	NA	NA	X = 1.985, df = 2, p = 0.371	NA	NA	NA

new-comer stops (duration)								
Habituation time			Mann-Whitney U-test				Mann-Whitney U-test	
			20-min vs 30-min				*20-min vs 30-min*	
rectangle			W = 41, p = 0.382				W = 34, p = 0.879	
round			W = 33.5, p = 0.916				W = 36, p = 0.721	
square			W = 46, p = 0.161				W = 41.5, p = 0.344	
Cage shape	Kruskal-Wallis rank sum test		Mann-Whitney U-test		Kruskal-Wallis rank sum test		Mann-Whitney U-test	
		rectangle vs round	*round vs square*	*rectangle vs square*		*rectangle vs round*	*round vs square*	*rectangle vs square*
20-min habituation	X = 1.085, df = 2, p = 0.581	NA	NA	NA	X =1.134, df = 2, p = 0.567	NA	NA	NA
30-min habituation	X = 2.375, df = 2, p = 0.305	NA	NA	NA	X = 0.965, df = 2, p = 0.617	NA	NA	NA

latency for first vocalization								
Habituation time			Mann-Whitney U-test				Mann-Whitney U-test	
			20-min vs 30-min				*20-min vs 30-min*	
rectangle			W = 23, p = 0.382				NA	
round			W = 43, p = 0.279				NA	
square			W = 31, p = 0.959				NA	
Cage shape	Kruskal-Wallis rank sum test		Mann-Whitney U-test		Kruskal-Wallis rank sum test		Mann-Whitney U-test	
		rectangle vs round	*round vs square*	*rectangle vs square*		*rectangle vs round*	*round vs square*	*rectangle vs square*
20-min habituation	X = 3.02, df = 2, p = 0.221	NA	NA	NA	NA	NA	NA	NA
30-min habituation	X = 3.255, df = 2, p = 0.196	NA	NA	NA	NA	NA	NA	NA

nb of vocalizations / min								
Habituation time			Mann-Whitney U-test				Mann-Whitney U-test	
			20-min vs 30-min				*20-min vs 30-min*	
rectangle			W = 24, p = 0.442				W = 23, p = 0.382	
round			W = 13.5, p = 0.059				W = 13, p = 0.050	
square			W = 33, p = 0.959				W = 31, p = 0.959	
Cage shape	Kruskal-Wallis rank sum test		Mann-Whitney U-test		Kruskal-Wallis rank sum test		Mann-Whitney U-test	
		rectangle vs round	*round vs square*	*rectangle vs square*		*rectangle vs round*	*round vs square*	*rectangle vs square*
20-min habituation	X = 3.528, df = 2, p = 0.171	NA	NA	NA	X = 5.013, df = 2, p = 0.082	NA	NA	NA
30-min habituation	X = 1.735, df = 2, p = 0.420	NA	NA	NA	X = 0.455, df = 2, p = 0.797	NA	NA	NA

mean duration of vocalizations								
Habituation time			Mann-Whitney U-test				Mann-Whitney U-test	
			20-min vs 30-min				*20-min vs 30-min*	
rectangle			W = 26, p = 0.574				W = 34, p = 0.879	
round			W = 25, p = 0.505				W = 29, p = 0.798	
square			W = 30, p = 0.879				W = 38, p = 0.574	
Cage shape	Kruskal-Wallis rank sum test		Mann-Whitney U-test		Kruskal-Wallis rank sum test		Mann-Whitney U-test	
		rectangle vs round	*round vs square*	*rectangle vs square*		*rectangle vs round*	*round vs square*	*rectangle vs square*
20-min habituation	X = 0.645, df = 2, p = 0.724	NA	NA	NA	X = 2.445, df = 2, p = 0.295	NA	NA	NA
30-min habituation	X = 0.035, df = 2, p = 0.983	NA	NA	NA	X = 0.095, df = 2, p = 0.954	NA	NA	NA
